# 

**Published:** 1886-11

**Authors:** 


Page

ORIGINAL COMMUNICATIONS:

A Case of Injury from Strong Electrical Current. H. N. Moyer ---- 429

A Visit to the Hospitals of Paris and to Pasteur's Laboratory. C. W. Earle ---- 432

Caries of the Right Parietal Bone. A. V. Park ---- 440

Hospital Practice in Vienna. F. Billings ---- 446

EDITORIAL:

A Possible New Field in Surgery ---- 454

The International Medical Congress 457

The Mississippi Valley Medical Association ---- 458

SOCIETY REPORTS:

*Chicago Medical Society; Meeting September 20.* The British Medical Association. N. S. Davis ---- 459

Pharyngeal and Nasal Surgery. W. E. Casselberry ---- 468

*Meeting October 4.* Hospitals of Paris and Laboratory of M. Pasteur. C. W. Earle ---- 475

Relations of Ametropia to Headache of Children. C. F. Sinclair 475

*Meeting October 18.* Alopecia Prematura. R. W. Bishop ---- 478

Page

Hospital Practice in Vienna. F. Billings ---- 480

The Hæmometer of Von Fleischl. F. S. Johnson ---- 482

*Chicago Gynæcological Society; Meeting August 20.* Twin Pregnancy, with One Placenta, etc., etc. W. W. Jaggard ---- 485

Discussion on Bartlett's Modification of Porro's Operation ---- 486

*Meeting September 17.* Supra-Vaginal Amputation of the Pregnant Uterus, etc. J. H. Etheridge ---- 494

Removal of the Uterus for Fibroids. C. T. Parkes ---- 506

*The American Academy of Medicine; Tenth Annual Meeting* ---- 517

BOOK REVIEWS:

Bright's Disease, etc. C. W. Purding 519

General Pathology. J. B. Sutton ---- 522

Principles and Practice of Surgery. F. H. Hamilton ---- 523

Manual of Dietetics, J. M. Fothergill ---- 525

Hand-Book of Practical Medicine. H. Eichhorst ---- 527

Drainage for Health. J. Wilson ---- 529

How We Treat Wounds To-Day. R. T. Morris ---- 532

